# PEPR DIADEM: Priority equipment and research program on the development of innovative materials using artificial intelligence

**DOI:** 10.1016/j.csbj.2024.09.019

**Published:** 2024-09-26

**Authors:** Fernando Lomello, Lucie Bard, Mario Maglione, Frédéric Schuster

**Affiliations:** a Université Paris-Saclay, CEA, DRF/IRFU/DIS, Gif-sur-Yvette 91191, France; b Université de Bordeaux, CNRS, Bordeaux INP, ICMCB, UMR 5026, Pessac F-33600, France; c Université Paris-Saclay, CEA, Cross-Cutting Program on Materials and Processes Skills, Gif-sur-Yvette 91191, France

**Keywords:** Artificial Intelligence (AI), Materials discovery, Sustainable materials, High-throughput synthesis, Digital simulation, Data-driven materials science

## Abstract

The quest to develop efficient, sustainable materials from non-critical, non-toxic resources is one of today's most formidable challenges in the current context of energy, transport, digital or healthcare transitions. In response, France launched the pioneering Priority Equipment and Research Program (PEPR) DIADEM in 2022. This innovative initiative, focused on DIscovery Acceleration for the Deployment of Emerging Materials (DIADEM), leverages Artificial Intelligence (AI) to accelerate the innovation chain from conception to realization, revolutionizing Materials Science sustainably. With a strategic emphasis on scientific synergy, PEPR DIADEM aims to expedite the discovery and development of novel materials essential for contemporary and future societal challenges. To achieve this, the program seeks to catalyze breakthroughs in areas ranging from energy efficiency to transportation, digitalization, and healthcare, covering a broad spectrum of materials from metallic alloys to functional nanostructures. Aligned with the Green Deal framework's ambitious targets, PEPR DIADEM addresses the urgent need for accelerated sustainable materials research. By utilizing cutting-edge technologies like rapid synthesis and characterization tools, automation, digital simulations, data management, AI, additive manufacturing, and thin film engineering, the program is set to significantly reshape the materials science landscape. As PEPR DIADEM embarks on its journey of innovation, it not only advances scientific knowledge but also holds the promise of addressing current global challenges and paving the way for a more sustainable and prosperous future.

## Highlights

• PEPR DIADEM accelerates sustainable material discovery with AI, reducing development time from 20 years to 4-10 years.
• PEPR DIADEM, led by CNRS and CEA, partners with 7 French universities and global collaborators to drive materials innovation.
• DIADEM employs cutting-edge MAPs for high-throughput synthesis, automated shaping, and tools for rapid material development.
• The program addresses energy, transport, and healthcare challenges, supporting the EU Green Deal and a sustainable future.
• DIADEM promotes interdisciplinary collaboration, training, and industry ties to ensure a lasting impact on materials science.

## Graphical Abstract

**Figure:**
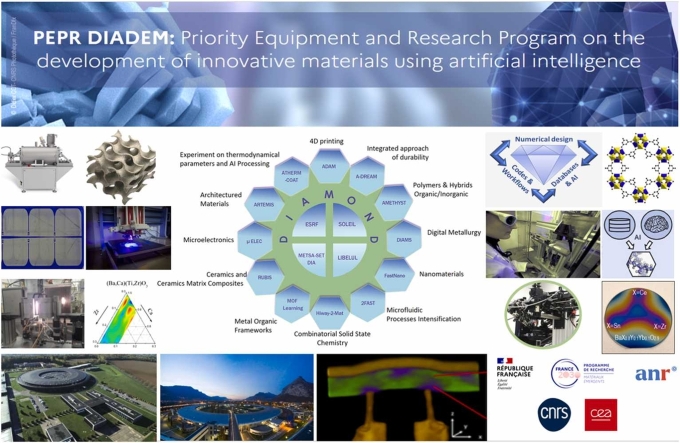

## 1 Description

Led by CNRS and CEA, in collaboration with seven academic partners — Université de Paris-Saclay, Sorbonne Université, Institut Polytechnique de Paris, Université Grenoble-Alpes, Université de Lorraine, Université de Bordeaux, and Université de Lyon 1 — the PEPR DIADEM aims to accelerate the discovery and integration of materials while addressing environmental and societal concerns. Endowed with a budget of €85 M by France 2030,^1^^,^^2^ the program seeks to design and market superior, sustainable materials, emphasizing AI. This funding establishes a network of four cutting-edge platforms across France, coordinated under the DIADEM DISCOVERY HUB. These platforms integrate high-throughput synthesis, combinatorial formulation, automated shaping, broadband characterization, and digital tools for multi-scale modelling, data mining, supervised learning, and AI adaptation.

The DIADEM Discovery Hub project builds on platforms initially dedicated to key material classes, crucial for accelerating the materials identification cycle from two decades to four to ten years.

These platforms encompass:

• Combinatorial and/or high-throughput synthesis and shaping of materials: Leveraging various robotized synthesis and additive manufacturing techniques to swiftly develop novel material compositions, including metallic, inorganic, and potentially bio-sourced polymer matrices. Thin film engineering plays a significant role in achieving desired performance outcomes, with special emphasis on synthesizing new architectured materials, composites, hybrids, and bio-inspired constructs.
• High-throughput chemical and structural characterization: Utilizing cutting-edge facilities such as the SOLEIL and ESRF synchrotrons, advanced Transmission Electron Microscopy (TEM), and fast chemical mapping methods such as Laser Induced Breakdown Spectroscopy (LIBS), to assess usage properties; in situ and *operando* characterizations are crucial for broadening and expediting data acquisition, particularly under extreme conditions.
• Digital simulation of materials and processes: Employing multiscale simulation tools, including AI approaches, seamlessly integrated into workflows to enable automated and high-throughput calculations.
• Databases for storage, management, and AI-driven exploitation: Structuring resulting data into databases and developing AI tools to build a digital infrastructure and enhance data exploitation, crucial for facilitating a productive dialogue between data and material sciences. The digital platform DIAMOND (green part of Fig. 2), bridging together the experiment-oriented platforms. This raises the challenge that all similar projects are facing, which is the necessary dialog between data and material sciences.

**Fig. 1:**
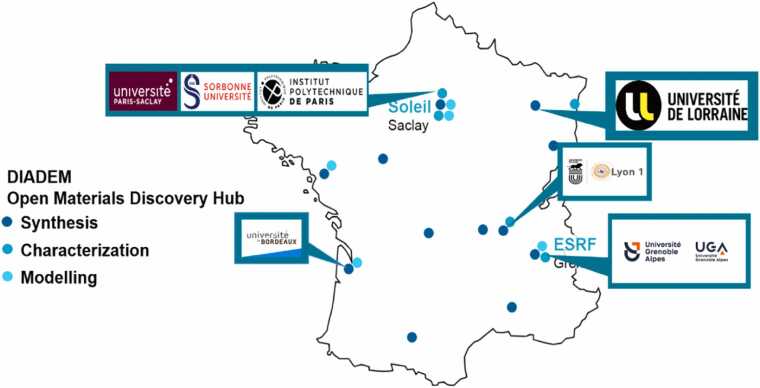
DIADEM Discovery hub & partner universities.

**Fig. 2:**
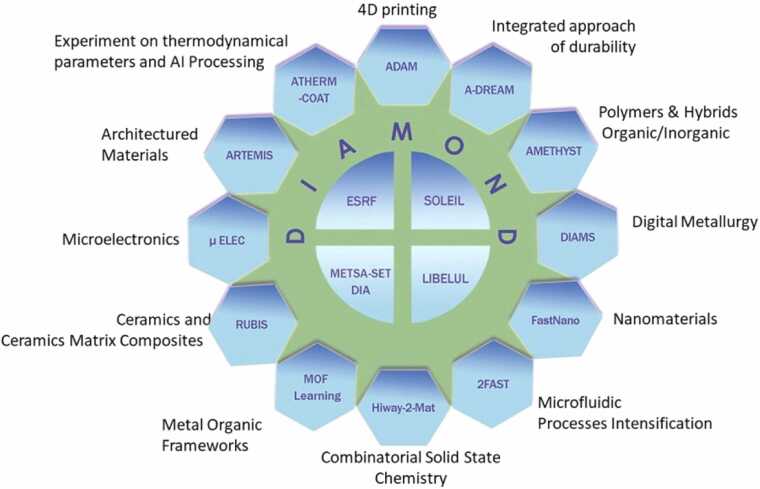
Actual seventeen targeted projects of PEPR DIADEM..^3^

Fig. 1 illustrates the distribution of platforms across the French territory that are part of the DIADEM DISCOVERY HUB, categorized by theme. The interplay among these platforms holds the potential to revolutionize innovation in materials science, driving sustainable solutions to address global challenges.

Seventeen targeted projects aim to build this unique network of platforms and to demonstrate its effectiveness in accelerating material discovery.

Two calls for proposals have been open to the extensive French materials community estimated of around 4000 researchers and engineers and lead to the selection of approximately 30 projects by an international panel. Interaction with existing infrastructures and research networks is a crucial criterion for project selection, and co-funding by companies and international partners is encouraged. The openness of the DIADEM DISCOVERY HUB to various partners will be a key indicator of its global success.

A dedicated training program for scientists, both novice and experienced, complements this framework, with international cooperation, including co-supervision of Ph. D. students and postdoctoral fellows, playing a vital role. International cooperation is central to DIADEM's mission, aligning with European initiatives such as IAM4EU and AMI2030.^4^

**Fig. 3:**
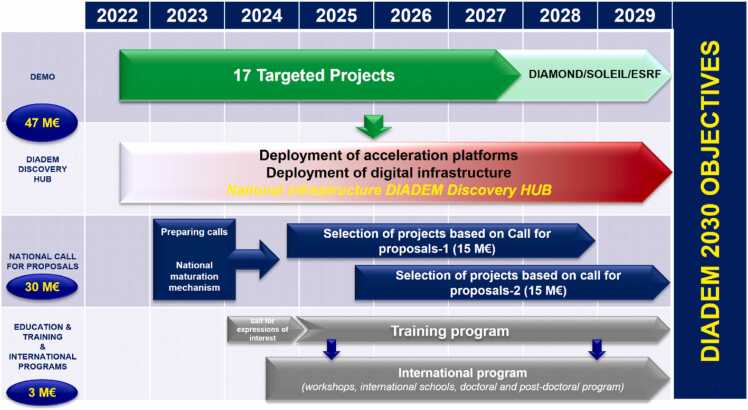
DIADEM global planning.

For the sake of clarity, Fig. 3 below illustrates the global plan for DIADEM through 2030.

## 2 Impact

Major innovations in the transition towards decarbonized energy, numerical transition, and quantum computing, as well as advancements in healthcare technologies, are intimately linked to the research and engineering of materials. In fact, materials not only present challenges in technological processes but often serve as the linchpin that determines progress, sustainability, economic feasibility, and ecological significance. New processing technologies such as 3D or 4D printing offer significant contributions to high-value industrial relocation and resilient supply chains in the post-Covid era. Artificial Intelligence (AI) is indispensable at all stages of materials processing: from modeling and design to synthesis, shaping, functional testing, and recycling. Throughout these steps, AI improves decision-making, thus accelerating the materials’ selection process and helping to lower environmental impact. Consequently, DIADEM's acceleration contributes to growth in the materials industry, benefiting both large companies and SMEs.

Public institutions worldwide, with connections to potential industrial transfers, actively support several ongoing projects. The "Material Genome Initiative" in the United States, for instance, has led to infrastructure developments in the field of AI for materials sciences.^5^ In Japan, combinatorial synthesis of materials started as early as 2000, particularly for functional thin films. Similar approaches are also underway in China and Germany. The European Open Science Cloud (EOSC) fosters an environment for hosting and processing research data to support EU science, with the DIADEM program committed to joining it.^6^ In Germany, the FAIRmat project shares similarities with DIADEM in objectives and implementation.^7^ Additionally, current discussions at the European level focus on the establishment of the 'Innovative Advanced Materials for EU' (IAM4EU) partnership, adopted in the second strategic plan for Horizon Europe 2025–2027.^8^ This initiative aims to create a collaborative Europe-wide research and innovation ecosystem to accelerate the development and market uptake of advanced materials. The IAM4EU partnership, supported by the Advanced Materials 2030 Initiative and the Graphene Flagship, will address the full innovation cycle of materials, from basic research to commercial products, aligning research investments with industrial needs and supporting a digital and circular economy.

The DIADEM program also entails a rapprochement with the "Innovation Platform MaterialDigital (PMD)," funded by the German Federal Ministry of Education and Research (BMBF), which is at the forefront of developing a robust infrastructure for the standardized digital representation of materials science and engineering. In collaboration with partners such as KIT, Fraunhofer IWM, FIZ, Leibnitz IWT, BAM, and MPIE, PMD is dedicated to establishing a comprehensive materials science data space.^9^

The exploratory nature and transformational capacity of DIADEM enable the sharing of experiences and knowledge. For example, the training of scientists, a key objective of DIADEM, benefits from significant subsidies. International schools, undergraduate, and doctoral programs can efficiently promote AI in materials science for young and experienced scientists. The program's opening to a large panel of possible industrial transfers enhances its potential for impactful outcomes. Direct involvement of industry stakeholders in the DIADEM Scientific Advisory Board aids in selecting materials under development with the most promise.

DIADEM's unique features and objectives include:

• Joint involvement of CEA, CNRS, and leading Universities, ensuring education, research, and initial steps of transfer to industry remain central throughout the program. This complementarity strengthens the program's foundations.
• Interdisciplinary Collaboration: DIADEM serves as a merger between chemists, physicists, and materials scientists, aiming for stronger interaction with experts in automation, databases, and AI. This collaboration involves approximately 4000 researchers and engineers across 200 laboratories, highlighting the program’s significance.
• Long-term Accessibility: The resulting infrastructure from DIADEM will be open to academic communities and companies beyond its lifetime. The commitment to accessibility and management of DIADEM platforms is crucial for its success.

**Fig. 4:**
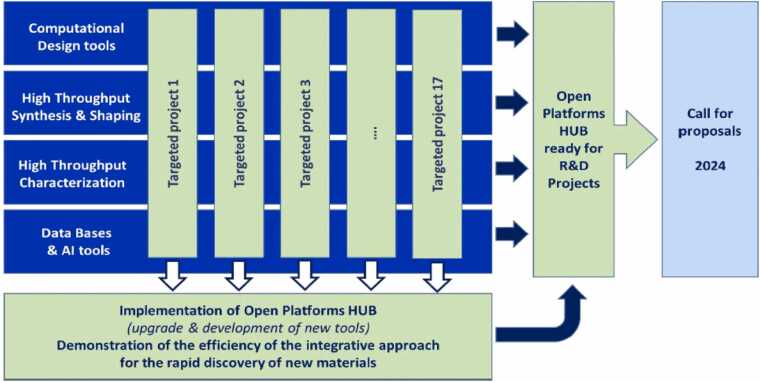
Role of the demonstrators in the implementation of DIADEM Open Platforms HUB and in the demonstration of an integrative approach.

Seventeen targeted projects (Fig. 4) have been defined to build a corpus of generic tools necessary for the success of this ambitious transformation program based on a balance between high-throughput synthesis and characterization, and the generation and exploitation of massive data flows by AI and simulation.

A college of experts from CNRS, CEA, and French reference universities in materials science and engineering have worked together to build the DIADEM architecture. The targeted projects are intended to implement the DIADEM DISCOVERY HUB, which will be open to the entire scientific community via the calls for proposals, and to prove the effectiveness of an integrated approach involving digital design, high-throughput synthesis, and characterization, together with data processing using AI to accelerate the discovery of new materials by a factor of 2 to 5 (from 20 years to 4–10 years).

The official launch of the PEPR took place on June 6, 2022, at the Maison de la Chimie in Paris; this was one of the first exploratory PEPR kick-off meetings.^10^ In 2023, efforts focused on initiating and ramping up these 17 targeted projects, including setting up platforms. Challenges related to the supply of spare parts and components impacted the reception of specific experimental equipment. However, all platform equipments are expected to be operational by September 2025, coinciding with the start of the projects selected in the second call for projects, which was launched in June 2024.

The first 17 targeted projects (Fig. 2) address specific aspects of materials science through diverse materials and advanced methodologies:

• DIAMOND (Data Integration and Modeling of Advanced Materials) is dedicated to developing a comprehensive digital infrastructure for accelerating materials development. By integrating databases, AI tools, and multi-scale modeling, DIAMOND enhances simulation and automation, utilizing machine learning for interatomic potentials to drive rapid, data-driven discoveries.,11,^12^
• ESRF (Accelerated X-ray Characterization of Materials at French CRG ESRF Beamlines) leverages the new Extremely Brilliant Source (EBS) for multi-scale material characterization. ESRF employs advanced diffraction and absorption spectroscopy to provide detailed insights into material structures and properties, ranging from atomic to macroscopic scales.,13,^14^
• METSA-SET-DIA (Mixing Electron Tomographies in Scanning mode with Advanced algorithms and Direct Electron Detectors) is focused on optimizing scanning electron tomography with direct electron detectors and deep learning algorithms. The project aims to improve tomography speed, automation, and precision through techniques such as EELS-SET, Nano-SET, and Ptycho-SET.,15,^16^
• SOLEIL (Platform for High Throughput Accelerated Materials Characterization at SOLEIL) is a targeted project advancing high-throughput materials characterization using beamlines like ANATOMIX and ANTARES. The DIADEM program within SOLEIL aims to develop new workflows that combine experimental data with AI for faster sample analysis and automated data processing.,17,^18^
• LIBELUL (LIBS for ELemental high throughpUt anaLysis) pioneers high-throughput elemental analysis using Laser-Induced Breakdown Spectroscopy (LIBS). This approach enables rapid, large-surface, and in-field analysis, complemented by Raman spectroscopy and luminescence, with AI enhancements for comprehensive data interpretation.,19,^20^
• ADAM (Accelerated Design of Architectured Materials) explores 3D and 4D printing technologies to create materials with tailored thermal and mechanical properties. By integrating 3D imaging, advanced numerical modeling, and AI, ADAM addresses energy and environmental challenges through innovative material design.,21,^22^
• A-DREAM (Accelerated Development of Corrosion-Resistant Materials) is focused on speeding up the discovery of corrosion-resistant materials. The project combines digital design, rapid synthesis, and corrosion testing, employing techniques such as combinatorial Physical Vapor Deposition (PVD) and Cold Spray to develop a range of materials and coatings.,23,^24^
• AMETHYST (AI and High Throughput Methods Guiding the Design of Polymeric Materials) aims at designing polymers with programmable degradability. By employing AI tools and high-throughput methods for preparation and characterization, AMETHYST seeks to create materials with customizable degradation profiles for diverse applications.,25,^26^
• DIAMS (Design by Artificial Intelligence and High Throughput Data of Advanced Alloys and Innovative Metallurgical Concepts for Structural Applications) accelerates the design of structural metallic alloys through high-throughput screening and characterization. Optimized by AI, DIAMS focuses on enhancing alloys with specific mechanical properties and performance criteria.,27,^28^
• FastNano (Low-Dimension Materials) develops AI-assisted synthesis reactors for high-throughput production and analysis of nanomaterials. The project emphasizes reactor validation and accessibility, facilitating rapid advancements in nanomaterial research.,29,^30^
• 2FAST (Federation of Fluidic Autonomous Labs to Speed-up Material Tailoring) designs autonomous microfluidic chips for miniaturized materials synthesis. Leveraging online data and machine learning, 2FAST aims to optimize chemical processes for precise and efficient material production.,31,^32^
• HIWAY-2-MAT (High-throughput Combinatorial and Autonomous Pathways in Solid State Chemistry) is focused on accelerating the discovery of new materials using autonomous high-throughput and combinatorial methods, with a particular emphasis on oxide materials. The project integrates advanced experimentation and data analysis to identify promising candidates.,33,^34^
• MOFsLearning (Accelerating the Design of MOFs through a Machine Learning-Assisted High-throughput Methodology) enhances Metal-Organic Framework (MOF) synthesis using AI-guided high-throughput methods, with a focus on applications in adsorption and catalysis.,35,^36^
• RUBIS (Data-driven Development and Fabrication of Ceramics and Thermostructural Composites: Research Platform on the Factory of the Future, Big Data, AI, and Associated Information Systems) is dedicated to developing thermal-structural ceramics and composites for extreme environments. RUBIS advances smart manufacturing techniques through digitization and machine learning.,37,^38^
• MicroElec (High Throughput Heterostructures Elaboration and Characterization for Microelectronic Applications) uses a high-throughput combinatorial approach assisted by AI to optimize microelectronic devices, including piezoelectric actuators and non-volatile memories.,39,^40^
• ARTEMIS (AcceleRaTed Discovery and dEvelopment of Smart Materials and Structures with 4D Printing TechnologieS) combines additive manufacturing with active materials to create smart objects that respond to external stimuli. It focuses on intelligent materials and structures with adaptive functionalities.,41,^42^
• ATHERM_COAT (Accelerated Thermodynamics and High-throughput Data for Optimization of Component Coatings for Energy Transition) develops tools for optimizing Chemical Vapor Deposition (CVD) and Atomic Layer Deposition (ALD) coatings, with the goal of enhancing performance and durability in energy transition technologies.,43,^44^

These 17 targeted projects collectively highlight the extensive diversity of materials and processes involved, from advanced characterization and new material design to process optimization and the integration of cutting-edge technologies like AI and additive manufacturing.

**Fig. 5:**
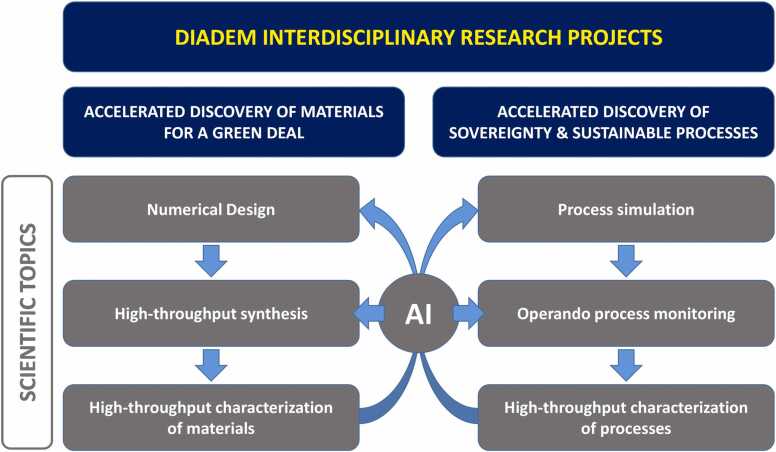
Interdisciplinary Research Projects (IRP) subjects of the calls for proposals.

The first DIADEM call for proposals was launched in April 2023 to promote a multidisciplinary approach through Interdisciplinary Research Projects (Fig. 5) of a methodological nature backed by major scientific themes largely consolidated by the French materials community. 45

The proposals must address one of the two following thematic axes:

• A thematic axis "Accelerated discovery of innovative and sustainable materials for a Green Deal" upstream of applications for major transitions (towards carbon-free energy and transport, towards frugal digital technology, towards the medicine of the future). These proposals should consolidate methodologies for the accelerated discovery of new families of materials of interest by coupling digital design, high-throughput synthesis, characterization, and data processing by artificial intelligence. These proposals must, as far as possible, include constraints of sobriety regarding the use of critical (or toxic) raw materials, which need to be substituted as much as possible.
• A thematic axis "Accelerating the mastery of synthesis and manufacturing processes" aimed at building or strengthening French technological sovereignty. The objective here will be to propose projects to accelerate the development of 1D, 2D, 3D, 4D processes, in particular by combining digital simulation approaches, in situ instrumentation and monitoring, and data processing by artificial intelligence. The idea is to move towards machines with increased autonomy and towards the digital twin of sovereignty processes.

Following the evaluation phase of the call for proposals 2023, only 4 of the 17 projects selected are carried out by laboratories already members of the targeted projects. 46 From the point of view of Universities not represented in DIADEM, we note the arrival of Strasbourg, Toulouse, Montpellier, Mulhouse, as selected project leaders. The involvement of the REALCAT platform of the University of Lille is also an important result of the call for proposals and will reinforce the network of platforms in DIADEM.^47^

For the construction of the second call of proposals in 2024, the criteria are more focused than in the first call of proposals, based on the mapping carried out thanks to the first call for proposals, the diversification of the themes supported will be continued.^48^ In addition to the new themes funded in the 2023 call for proposals (catalysis, molecular materials, ionic liquids, photovoltaics, magnetic materials, bio-sourced foams, coatings, and thin films), additional themes will be specifically targeted: thermoelectricity, materials for spintronics, advanced materials for future medical technologies. One or two projects may be selected on each of these themes based on the projects submitted and the evaluation by the sovereign international committee.

Several evaluation criteria have been defined for the 2024 call for proposals:

• The use of DIADEM platforms must be systematized and widely explained.
• The use of artificial intelligence methodologies and tools will be described in detail.
• Even if not obligatory, the positioning of projects at the interface with other PEPRs will be an additional evaluation criterion.
• Internationalization: Consortia will be able to integrate international partners who are not funded but who can, for example, co-fund doctoral or post-doctoral fellowships or welcome students and permanent researchers.
• Industrial partnerships: The transfer of disruptive innovations to the socio-economic world will be one of DIADEM's indicators of success. The integration of unfunded companies is therefore encouraged.

## 3 Results

Regarding the establishment of platforms, the pioneering large-scale experimental device fully funded by DIADEM was the SAXS, installed at the NIMBE laboratory (a joint unit of CEA/CNRS Saclay). This equipment enables real-time monitoring of high-throughput nanoparticle synthesis in liquid mediums. Notably, a publication on this subject was already released in 2024 [1].

DIADEM's targeted projects have yielded significant scientific advancements, as evidenced by 16 published articles. Notable achievements include:

• LIBELUL: Pioneering high-throughput LIBS imaging and neural network-based analysis [2], [3], [4], [5]
• FastNano: Quasi van der Waals Epitaxy of Rhombohedral-Stacked Bilayer WSe₂ on GaP (111) Heterostructure, the stacking order and electronic band structure of trilayer WSe_2_ films, and the diameter and chirality of natural and synthetic imogolite [1], [6], [7], [8]
• ESRF: Identifying structural fragility zones in Li-ion batteries, a critical factor for improved performance [9], [10]
• MOFsLearning: Developing innovative models for adsorption processes in porous materials. [11], [12]
• HIWAY-2-MAT: Pioneering autonomous and combinatorial exploration methods in solid-state chemistry [13].
• ARTEMIS: Developing new 4D Printed enhanced structures [14], [15]
• SOLEIL: Making significant contributions to understanding oscillatory buckling reversal in magnetic textures [16].

Furthermore, it is worth mentioning the active participation of DIADEM management in a European consortium's publication emphasizing the significance of Materials Acceleration Platforms [17].

To ensure a sustainable pipeline of AI-savvy materials scientists, PEPR DIADEM has established a comprehensive training program. This program focuses on:

• Foundations of AI: Providing a solid understanding of AI fundamentals and their applications in materials science.
• Continuous Learning: Offering ongoing workshops and specialized courses to keep researchers updated on the latest developments.
• International Collaboration: Fostering partnerships and research exchanges to broaden perspectives and attract top talent.

The training program is scheduled to launch in 2025, providing researchers with the opportunity to acquire the skills necessary to drive innovation in materials science.

By investing in training and fostering innovation, PEPR DIADEM is poised to drive transformative advancements in materials research and address critical societal challenges.

## 4 Discussion

The PEPR DIADEM program has made significant advancements in the rapid discovery and development of innovative materials by leveraging the synergy between AI and materials science. The ongoing establishment of a nationwide network of platforms for high-throughput synthesis, characterization, and digital simulation is anticipated to effectively reduce the innovation cycle time from decades to mere years. This accelerated timeline is crucial for addressing current societal challenges in energy, healthcare, transportation, and digitalization.

The integration of AI into materials science will enable more efficient data management, predictive modeling, and decision-making processes. These advancements have resulted in the identification of new material compositions with enhanced performance characteristics, which are critical for developing next-generation technologies. The program’s emphasis on sustainability and the use of non-critical, non-toxic resources aligns with global initiatives aimed at minimizing environmental impact and promoting circular economies.

The outcomes of the PEPR DIADEM program align closely with global initiatives such as the Materials Genome Initiative in the United States, which similarly focuses on expediting materials discovery through the application of digital tools and AI. Moreover, the program’s methodology resonates with the combinatorial synthesis methods developed in Japan and the AI-driven materials screening processes in China and Germany. These comparisons underscore the international trend towards integrating AI in materials science to achieve faster and more cost-effective innovations.

In the European context, the DIADEM program also aligns with the "Innovation Platform MaterialDigital (PMD)," funded by the German Federal Ministry of Education and Research (BMBF). Additionally, the European Open Science Cloud (EOSC) and the FAIRmat project in Germany share similar objectives with DIADEM, emphasizing the importance of data sharing, interoperability, and AI-driven research. DIADEM's collaborative efforts with these initiatives further strengthen its impact and potential for fostering European and international cooperation in the field.

Despite PEPR DIADEM’s strategy, the program encounters several challenges. One major challenge is the cultural gap between materials scientists and AI experts, which can impide effective collaboration and integration of interdisciplinary knowledge. Bridging this gap necessitates ongoing efforts in education and training, as well as fostering a collaborative research environment. To address this challenge, DIADEM plans to develop a high-quality training programme, starting in 2025, which will complement existing initiatives in three areas: initial training, continuing education and doctoral and post-doctoral training.

Furthermore, DIADEM will collaborate with the recently established "AI for sciences and sciences for AI" center by CNRS.^49^

Additionally, the program’s reliance on existing infrastructures such as synchrotron facilities and advanced characterization tools may limit its scalability. Expanding access to these resources and developing decentralized platforms could enhance the program's reach and impact.

Future directions for the PEPR DIADEM program include:

• Enhanced AI Integration: Further development of AI algorithms tailored to materials science applications, including more sophisticated predictive models and machine learning techniques, will improve the accuracy and efficiency of materials discovery.
• Expanded Collaboration: Strengthening international partnerships and fostering collaborations with industry stakeholders will enhance the program’s capacity to translate research findings into practical applications and commercial products.
• Broadening Training Programs: Implementing comprehensive training programs in AI and materials science for both novice and experienced researchers will address the skills gap and promote interdisciplinary expertise.
• Sustainable Practices: Continued emphasis on sustainability will lead the program to explore innovative recycling and upcycling methods for materials, as well as developing bio-inspired and eco-friendly materials.

## 5 Conclusion

In summary, the PEPR DIADEM program represents a significant advancement in the field of materials science by leveraging artificial intelligence (AI) to facilitate the accelerated discovery and development of advanced materials. The establishment of a nationwide network of platforms integrating high-throughput synthesis, characterization, and digital simulation, the program is believe to promote the progress in minimizing the innovation cycle for materials research.

Key takeaways from the program include:

• Interdisciplinary Collaboration: The initiative fosters the collaboration between academic institutions, research organizations, and industry partners, demonstrating the importance of interdisciplinary cooperation in enhancing scientific innovation.
• AI-Driven Discovery: By harnessing AI algorithms and data-driven approaches, PEPR DIADEM has identified novel materials with enhanced properties, thereby enabling significant breakthroughs across various sectors, including energy, healthcare, transportation, and digitalization.
• Sustainability Focus: With a strong emphasis on sustainability and the use of non-critical, non-toxic resources, the program aligns with global initiatives designed to mitigate environmental challenges and promote circular economies.
• Practical Applications: The advancements achieved through PEPR DIADEM exhibit considerable potential for practical applications, including the development of more efficient energy storage systems, innovative medical devices and sustainable manufacturing processes.
• Innovation Potential: Future directions for the program highlight the substantial potential for innovation in materials science, driven by AI-enabled technologies, collaborative research initiatives, and strategic investments.

As the global community addresses current challenges like climate change, resource depletion, and the demand for sustainable energy solutions, initiatives such as PEPR DIADEM represent a crucial advancement in the development of innovative materials that support sustainability goals and foster economic resilience.

## CRediT authorship contribution statement

**Fernando Lomello:** Writing – review & editing, Writing – original draft, Visualization, Validation, Methodology, Conceptualization. **Lucie Bard:** Writing – review & editing, Supervision. **Mario Maglione:** Writing – review & editing, Supervision. **Frédéric Schuster:** Writing – review & editing, Supervision.

## Declaration of Competing Interest

The authors declare that they have no known competing financial interests or personal relationships that could have appeared to influence the work reported in this paper.

## Footnotes

11 https://anr.fr/ProjetIA-22-PEXD-0015

12 https://www.pepr-diadem.fr/projet/diamond-en/

13 https://anr.fr/ProjetIA-22-PEXD-0011

14 https://www.pepr-diadem.fr/projet/esrf-en/

15 https://anr.fr/ProjetIA-22-PEXD-0013

16 https://www.pepr-diadem.fr/projet/metsa-set-dia-en/

17 https://anr.fr/ProjetIA-22-PEXD-0012

18 https://www.pepr-diadem.fr/projet/soleil-en/

19 https://anr.fr/ProjetIA-22-PEXD-0014

20 https://www.pepr-diadem.fr/projet/libelul-en/

21 https://anr.fr/ProjetIA-22-PEXD-0002

22 https://www.pepr-diadem.fr/projet/adam-en/

23 https://anr.fr/ProjetIA-22-PEXD-0003

24 https://www.pepr-diadem.fr/projet/a-dream-en/

25 https://anr.fr/ProjetIA-22-PEXD-0004

26 https://www.pepr-diadem.fr/projet/amethyst-en/

27 https://anr.fr/ProjetIA-22-PEXD-0005

28 https://www.pepr-diadem.fr/projet/diams-en/

29 https://anr.fr/ProjetIA-22-PEXD-0006

30 https://www.pepr-diadem.fr/projet/fastnano-en/

31 https://anr.fr/ProjetIA-22-PEXD-0007

32 https://www.pepr-diadem.fr/projet/2fast-en/

33 https://anr.fr/ProjetIA-22-PEXD-0008

34 https://www.pepr-diadem.fr/projet/hiway-2-mat-en/

35 https://anr.fr/ProjetIA-22-PEXD-0009

36 https://www.pepr-diadem.fr/projet/moflearning-en/

37 https://anr.fr/ProjetIA-22-PEXD-0010

38 https://www.pepr-diadem.fr/projet/rubis-en/

39 https://anr.fr/ProjetIA-22-PEXD-0018

40 https://www.pepr-diadem.fr/projet/microelec-en/

41 https://anr.fr/ProjetIA-22-PEXD-0016

42 https://www.pepr-diadem.fr/projet/artemis-en/

43 https://anr.fr/ProjetIA-22-PEXD-0017

44 https://www.pepr-diadem.fr/projet/atherm_coat-en/

## Footnotes

3 https://www.pepr-diadem.fr/en/

## Footnotes

1 https://anr.fr/fr/france-2030/france-2030/

2 https://www.enseignementsup-recherche.gouv.fr/sites/default/files/2022–07/dossier-de-presse---france-2030---les-laur-ats-de-la-seconde-vague-d-appels-projets-des-pepr-19288_1.pdf

4 https://www.ami2030.eu/

5 https://www.mgi.gov/

6 https://research-and-innovation.ec.europa.eu/strategy/strategy-2020–2024/our-digital-future/open-science/european-open-science-cloud-eosc_en

7 https://www.fairmat-nfdi.eu/fairmat/

8 https://www.ami2030.eu/events/mar-13-info-session-on-the-proposed-eu-partnership-under-horizon-europe-innovative-advanced-materials-for-europe/

9 https://www.materialdigital.de/

10 https://www.inc.cnrs.fr/fr/cnrsinfo/diadem-retour-sur-le-lancement-du-pepr-dedie-aux-materiaux-emergents

45 https://anr.fr/fr/france-2030/france2030/call/pepr-diadem-appel-a-projets-dispositifs-integres-pour-lacceleration-du-deploiement-de-materiaux/

46 https://www.pepr-diadem.fr/calls-for-proposals-2/call-for-proposals-2023/

47 https://www.realcat.fr/en/home/

48 https://www.pepr-diadem.fr/call-2024/

49 https://aissai.cnrs.fr/
